# New Treatment for Tape-Worm

**Published:** 1873-11

**Authors:** J. H. Bill

**Affiliations:** Surg. U. S. A.


New Treatment for Tape-Worm.—*J. H. Bill, Surg. U. S. A.*—The idea then struck me, if small quantities of "carbolic acid" are so pernicious to leeches, is it not probable that the drug might be used against a tape-worm. Accordingly, having purged the patient, I administered six grains of "carbolic acid" in a half-pint of water, four times a day. After two days of this treatment, as only a few joints of the worm had been voided, I changed the vehicle from a liquid to a pill form. Powdered extract of liquorice was used, and each five-grain pill contained two grains of "carbolic acid;" of these the man took one every hour, and a purge of rhubarb and jalap every morning. He soon began to pass large fragments of the worm, and on the third day the head and about four feet of the body of a tænia solium. He had taken thirty-five pills without inconvenience of any kind. He got well quickly, has remained well since, and I am satisfied is cured. I have since thought that the pills would have been improved by a delicate coating of paraffine; the object being, of course, to cause the pill to pass through the stomach unchanged into the intestine, carrying with it the "carbolic acid" in direct contract with the worm.

